# From Gut Commensal to Opportunistic Pathogen: A Narrative Review of *Butyricimonas* Infections in Humans

**DOI:** 10.3390/antibiotics15030297

**Published:** 2026-03-14

**Authors:** Afroditi Ziogou, Alexios Giannakodimos, Ilias Giannakodimos, Andreas G. Tsantes, Stella Baliou, Dimitrios Rigopoulos, Petros Ioannou

**Affiliations:** 1 Department of Internal Medicine, 417 Army Equity Fund Hospital, 11521 Athens, Greece; 2 Department of Cardiology, Tzaneio General Hospital of Piraeus, 18537 Piraeus, Greece; 3 Departmentof Urology, Attikon General Hospital of Athens, 12462 Athens, Greece; 4 Laboratory of Haematology and Blood Bank Unit, “Attiko” Hospital, School of Medicine, National and Kapodistrian University of Athens, 11527 Athens, Greece; 5 Microbiology Department, “Saint Savvas” Oncology Hospital, 11522 Athens, Greece; 6 Department of Internal Medicine, University Hospital of Heraklion, 71003 Heraklion, Greece

**Keywords:** *Butyricimonas*, infection, meningitis, bloodstream, peritonitis, endocarditis, pneumonia, osteomyelitis

## Abstract

Background/Objectives: *Butyricimonas* species constitute a genus of Gram-negative, anaerobic bacteria that are part of the human gut microbiota. Infections caused by these organisms are extremely rare in clinical practice. While uncommon in the general population, their occurrence is higher among immunocompromised individuals or patients with significant underlying health conditions. This review aims to compile and analyze all reported cases of human *Butyricimonas* infections, focusing on epidemiology, microbiological characteristics, antimicrobial resistance patterns, treatment strategies, and associated mortality. Methods: This review was conducted using data retrieved from the PubMed/MEDLINE and Scopus databases. Results: A total of 14 publications described *Butyricimonas* infections affecting 14 patients. The mean age of those affected was 66.46 years, and 10 (71.4%) were male. The most frequently reported predisposing factor was a history of malignancy, observed in almost one-third of cases (30.8%). Clinically, fever, organ dysfunction, and shock were the most common presentations (fivecases), followed by sepsis and the need for ICU in fourpatients. In vitro studies indicated that the isolates were generally susceptible to carbapenems and metronidazole, with only high resistance levels observed to penicillin. Among the antimicrobial therapies used, carbapenems were the most commonly administered (50%), followed by piperacillin/tazobactam (41.7%) and metronidazole (33.3%). The overall mortality rate across the cohort was 16.7%, with infection-attributable deaths representing 8.3% of cases. Conclusions: Given the potential of *Butyricimonas* species to cause severe infections, clinicians should consider this organism in patients presenting with unexplained bacteremia or intra-abdominal infections, particularly in the setting of mucosal disruption or immune dysfunction.

## 1. Introduction

*Butyricimonas* spp. is a strictly anaerobic, non-pigmented, Gram-negative bacillus belonging to the family *Porphyromonadaceae*. Since its initial isolation from rat fecal samples in 2009 and subsequent taxonomic classification based on 16S rRNA gene sequencing, the genus *Butyricimonas* has expanded to include several species, currently comprising *B. virosa*, *B. synergistica*, *B. paravirosa*, *B. faecihominis*, *B. phoceensis*, *B. faecalis*, and *B. vaginalis* [1,2,3]. Although primarily regarded as a commensal organism, *Butyricimonas* spp. has increasingly been recognized as a potential human pathogen, highlighting its clinical relevance in specific patient populations. Clinically significant infection caused by *Butyricimonas* species remains exceptionally uncommon, with only sporadic cases reported in the literature. Nevertheless, the emergence of invasive infections underscores the capacity of this genus to transition from commensalism to pathogenicity under certain conditions. These organisms are considered opportunistic pathogens, primarily affecting individuals with underlying conditions such as malignancy, particularly colorectal cancer, hematological disease, immunosuppression, recent gastrointestinal or gynecological surgery, or advanced age [4,5]. In this context, disruption of mucosal barriers and immune dysfunction appear to play a critical role in facilitating bloodstream invasion. Anaerobic bacteraemia, which constitutes approximately 1–17% of all bloodstream infections, typically arises from endogenous sources, underscoring the pathogenic potential of communal anaerobes under favorable conditions [6]. From a diagnostic perspective, *Butyricimonas* infections pose significant challenges as definitive identification requires advanced molecular techniques, most notably 16S rRNA gene sequencing, due to limitations of conventional diagnostic methods. This diagnostic complexity likely contributes to under-recognition and under-reporting of infections caused by this genus. At present, there are no established treatment guidelines for *Butyricimonas* infections, and clinical management relies largely on empirical antimicrobial therapy guided by susceptibility testing. Reported mortality rates are low and appear to be influenced by both the anatomical site of infection and the patient’s underlying clinical status.

This review synthesizes all reported cases of *Butyricimonas* spp. infections in humans, with particular emphasis on their epidemiological characteristics and susceptibility patterns. In addition, it examines the microbiological features of these microorganisms, therapeutic management strategies, and patients’ clinical outcomes. By critically appraising the existing literature, this work also aims to identify current knowledge gaps, especially with regard to predisposing risk factors and optimal treatment approaches, and to contribute to a more comprehensive understanding of this emerging pathogen.

## 2. Results

### 2.1. Included Studies’ Characteristics

**Figure 1:**
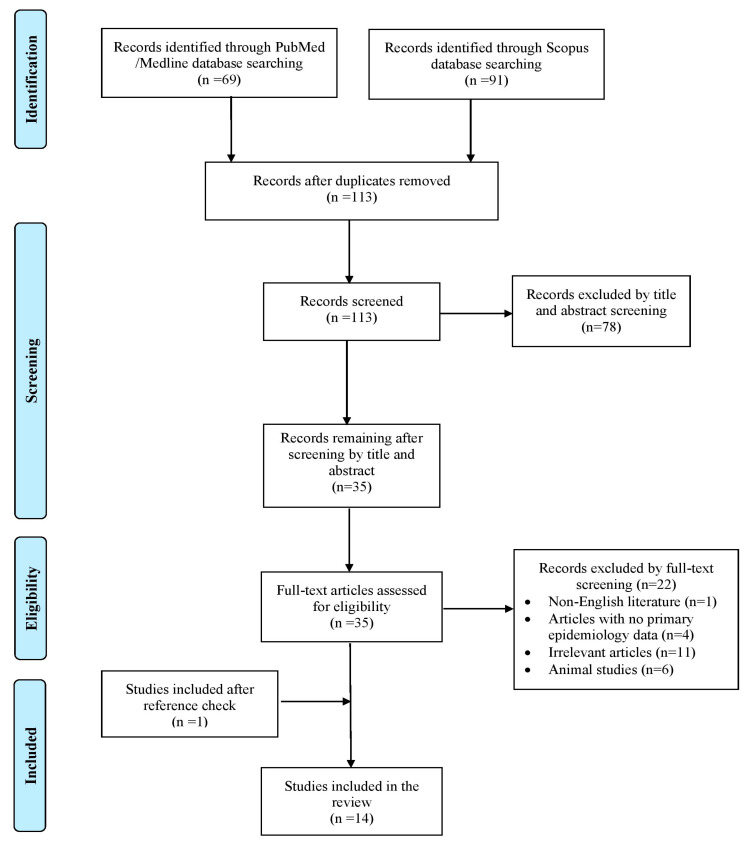
Trial Flow of this Review.

Database searches in PubMed/Medline and Scopus yielded 160 records. Following removal of duplicate articles, title and abstract screening, full-text assessment, and additional reference tracking, 14 publications fulfilled the inclusion criteria and were retained for analysis. These studies collectively reported 14 individual cases [3,4,7,8,9,10,11,12,13,14,15,16,17,18]. A schematic overview of the selection procedure is provided in Figure 1. Of the documented cases, eight (57.1%) were reported from Europe, three cases (21.4%) from Asia, and three additional cases (21.4%) from the Americas, respectively. All eligible publications were limited to individual case report studies. Table S1 presents the characteristics of all included studies.

### 2.2. Epidemiology of Butyricimonas *spp.* Infections

The average age of patients affected by *Butyricimonas* spp. infections was 66.46 years, spanning a minimum of 38 and a maximum of 94 years. Among the 14 reported cases, males accounted for 10 patients (71.4%). Concerning risk factors, a history of malignancy was identified in four cases (30.8%), three of which involved the gastrointestinal tract, while one involved breast cancer. Active malignancy was recorded in three different individuals (23%); in this subgroup, two malignancies were located in the gastrointestinal tract, specifically in the colon and duodenum; the third was a case of prostate cancer with affected lymph nodes. Three patients (23%) had a documented history of a recent surgical procedure, which was defined as surgery performed within the preceding three months. Among this patient group, patient ages ranged from 72 to 81 years. Bloodstream infection was the predominant clinical presentation in this group, occurring in two cases, while one patient developed necrotizing fasciitis. Mortality in diabetic patients was limited to a single case, with the remaining two achieving full recovery.

Obesity was present in two patients (15.4%); one patient developed chronic bone and joint infection, while the second developed a gastrointestinal infection. Clinical cure and survival were documented for one of these two patients; no data on clinical outcome are available for the second patient. Other predisposing, less frequently described conditions included immunosuppression in two cases (15.4%), end-stage renal disease (ESRD) on peritoneal dialysis, type 2 diabetes mellitus (T2DM), and trauma, each identified in one individual (7.7%). Of note, in four individuals (30.8%), no predisposing factors were identified. Amongst the 14 included studies, there was one study that did not provide data on risk factors. An overview of demographic features and clinical characteristics across all cases is summarized in Table 1.

### 2.3. Clinical Manifestations of Butyricimonas *spp.* Infections

Bacteremia was the most frequent manifestation, identified in eight patients (57.1%), while gastrointestinal infection occurred in five patients (35.7%). Among these fivecases, four individuals exhibited gastrointestinal infection in combination with bacteremia. Skin and soft tissue involvement was reported in three patients (21.4%), and peritonitis due to *Butyricimonas* spp. was observed in another three cases (21.4%). In two out of the three cases of peritonitis, bloodstream infection was present. Additionally, amongst the three cases with skin or soft tissue infections, one developed bacteremia. Bone and joint infection, as well as vaginal infection, were each documented in one patient (7.1%), and in these individuals, bacteremia was not observed. Notably, no cases of respiratory infection, meningitis, or endocarditis were reported. Severe invasive infection was described in 11 patients (78.6%), while three individuals (21.4%) exhibited only local infection. All reported fatalities in this review occurred exclusively in patients with invasive infection.

Fever, organ dysfunction, and shock were the most commonly reported clinical manifestations of *Butyricimonas* infections, each occurring in five patients (5/10, 50% cases of fever and 5/12, 41.7% cases of organ dysfunction and shock, respectively). In two out of the 14 cases, clinical complications were not reported. All individuals who developed fever had underlying bacteremia. Three out of five cases of organ dysfunction consisted of multi-organ failure, while four out of five cases of shock included septic shock. The remaining case of shock was due to hypovolemia resulting from gastrointestinal bleeding. Specific associations between infection site and fever, shock, and organ dysfunction are depicted in Table 2. Sepsis was present in four cases (33.3%), all of which developed septic shock. Need for intensive care unit (ICU) was documented in four patients (33.3%); in all these cases, septic shock was also present. Abscess formation occurred in two patients (16.7%), while the need for haemodialysis occurred in one individual (8.3%). Disease recurrence was noted only in one patient (8.3%). Symptom duration varied widely, ranging from immediate onset to seven days prior to hospital admission.

### 2.4. Antimicrobial Resistance and Microbiology of Butyricimonas *spp.* Infections

*Butyricimonas* spp. was most frequently isolated from blood cultures, accounting for ninecases (64.3%). Less commonly, the organism was recovered from stool, dialysate, or intra-operative specimens’ cultures (one case, 7.1% respectively), while wound and vaginal swabs were positive in one case each (7.1%). Among the recovered isolates, eight were identified as *Butyricimonas virosa* (61.5%), which emerged as the dominant species. Two cases involved *B. faecihominis* infection (15.4%), while *B. phoceensis*, *B. paravirosa,* and *B. vaginalis* were each isolated once in different cases (7.7%). Polymicrobial disease was documented in five patients (35.7%), with concurrent isolation of *Eubacterium callanderi*, *Brachyspira pilosicoli*, *Finegoldia magna*, *Bacteroides vulgatus*, *Clostridium tertium*,and *Atopobium massiliense*. Identification of the causative organisms relied predominantly on molecular diagnostic techniques; 16s rRNA alone was used to confirm the pathogen in four cases (30.8%). Matrix-assisted laser desorption/ionization time-of-flight mass spectrometry (MALDI-TOF MS) and genome sequencing contributed to species-level identification in three (23.1%) and two additional cases (15.4%), whereas a combination of MALDI-TOF MS and 16s rRNA was employed in three instances (23%). Finally, a combination of VITEK 2 and MALDI-TOF MS was applied in one case (7.7%) to successfully identify the pathogen.

Antimicrobial susceptibility testing was performed for 10 patients (71.4%). Susceptibility was evaluated with Etest in 7/9 cases (77.8%), while the disk diffusion method and application of EUCAST criteria were applied in one case, respectively (11.1%). In 1/10 cases, the method applied to test the pathogen’s susceptibility profile is not reported. Comprehensive antibiotic resistance data, including the number of isolates tested against each agent and the corresponding resistance rates, are summarized in Table 3. Each isolate was evaluated using a distinct panel of antimicrobial drugs. Universal susceptibility was observed for aminopenicillins with β-lactamase inhibitors and carbapenems. Specifically, all isolates tested against carbapenems (7/7) and aminopenicillins with β-lactamase inhibitors (4/4) demonstrated susceptibility, as did the isolates assessed for teicoplanin (1/1), rifampicin (1/1), and Trimethoprim-sulfamethoxazole (TMP-SMX) (1/1).

### 2.5. Treatment and Outcome of Butyricimonas *spp.* Infections

Antimicrobial therapy was administered to 11 patients; one patient did not receive antibiotics, while in two cases, it is not reported whether or not antibiotics were administered. Carbapenems were the most commonly used agents, given to 6/12 patients (50%), followed by piperacillin/tazobactam in 5/12 cases (41.7%) and metronidazole in four cases (33.3%). Cephalosporins were prescribed in 3/12 patients (25%). Colistin, vancomycin, quinolones, aminoglycosides, clindamycin, and TMP-SMX were each used in 1/12 patients (8.3%). Surgical intervention alongside antibiotic therapy was performed in seven patients (58.3%), with the procedure tailored to the infected organ. For appendicitis cases, this typically included appendectomy, whereas abscesses were managed with drainage. Among survivors, the mean duration of antimicrobial treatment was 29 days, ranging from one week for a patient with terminal ileitis and bacteremia to three months for a patient with severe bone infection.

The overall mortality among the 14 patients was 16.7% (2/12, since in two cases clinical outcome was not reported), with *Butyricimonas* infection specifically responsible for 8.3% of deaths (1/12); in the other individual, *Acinetobacter* septicaemia was the cause of death. Both deceased patients had multiple risk factors that likely contributed to severe disease, and both developed bacteremia. One of these patients developed a skin and soft tissue infection. Both exhibited shock and were admitted to the ICU; in the first patient, the shock was of septic origin, while in the second, it was due to hypovolemia resulting from severe bleeding due to diffuse esophageal ischemia. Carbapenems were administered to both individuals.

## 3. Discussion

To our knowledge, this represents the first narrative review to synthesize the available literature on the identification and clinical management of *Butyricimonas* spp. infections. Data have been collected from published studies describing human infections caused by *Butyricimonas* species, providing an integrated overview of their epidemiology, microbiological characteristics, clinical presentations, therapeutic approaches, and patient outcomes. The synthesis of these findings enables a more complete understanding of the pathogen’s epidemiology and potential clinical impact, aspects that have remained fragmented in prior reports. Bloodstream and gastrointestinal tract infections emerged as the most frequently reported clinical syndromes, while *Butyricimonas virosa* was identified as the predominant species. Carbapenems constituted the most commonly administered antimicrobial agents in the available cases. Notably, the analysis highlights a relatively low mortality rate associated with *Butyricimonas* infections.

Owing to the limited number of *Butyricimonas* spp. infections reported to date, the delineation of a robust epidemiological profile remains challenging [9]. Diagnosis of this rare entity is highly dependent on clinical suspicion, underscoring the importance of heightened awareness among clinicians. In this review, most documented cases occurred in male patients, with a mean age of 66.46 years. Notably, the majority of cases were reported from Europe, followed by the United States and Asia. No cases were reported from African countries or Oceania. The predominance of reported cases from Europe is best explained by reporting and detection biases, rather than true geographic epidemiology. European centers, particularly tertiary hospitals and reference laboratories, more frequently use 16S rRNA gene sequencing and MALDI-TOF MS, which are essential for identifying rare anaerobes such as *Butyricimonas*. In regions such as Asia, where these tools are less routinely available, infections may remain unidentified or be misclassified as unspecified anaerobic Gram-negative bacilli [19,20]. Moreover, European clinicians and microbiologists may be more inclined to publish rare or unusual infections, especially anaerobic bacteraemia cases, leading to a disproportionate representation in the literature. Several European laboratories have longstanding expertise in anaerobic bacteriology and taxonomic research, including studies on the *Porphyromonadaceae* family, thus increasing the likelihood of recognizing and reporting novel or uncommon species [21]. Despite the presence of isolated reports, the scarcity of global studies precludes the establishment of a definitive epidemiological understanding of *Butyricimonas* infections.

*Butyricimonas* spp., initially isolated from rat feces in 2009, is classified within the *Porphyromonadaceae* family. It is a Gram-negative, short rod-shaped bacterium characterized by its non-motile and non-spore-forming nature. It lacks both catalase and oxidase activities and exhibits strict anaerobic growth, with an optimal temperature of 37 °C. The predominant cellular fatty acid is 13-methyl-tetradecanoic acid, accounting for approximately 75% of the total lipid composition. Colonies of *Butyricimonas* appear opalescent and beige with regular margins, reflecting their distinctive phenotypic profile [3]. To date, the genus *Butyricimonas* comprises seven recognized species: *B. virosa*, *B. synergistica*, *B. paravirosa*, *B. faecihominis*, *B. phoceensis*, *B. faecalis,* and *B. vaginalis*, with *B. virosa* being the only species reported to exhibit pathogenic potential [1,9,16,22]. *B. virosa* was also the predominant causative pathogen in most cases of infection. Members of the genus *Butyricimonas* are recognized constituents of the normal human intestinal microbiota [23,24]. However, invasive infections caused by these bacteria have been documented, including bacteremia, peritonitis, gastrointestinal tract infections, and bone infections. Malignancy has been identified as a significant predisposing factor for infection, although cases in immunocompetent individuals have also been reported [15,17].

*Butyricimonas* species exhibit a largely saprophytic nature but have demonstrated pathogenic potential under specific host-dependent conditions. These anaerobic, Gram-negative bacilli are part of the normal human gut microflora, where they are generally associated with short-chain fatty acid production and intestinal homeostasis [4,25]. *Butyricimonas* produces butyrate, a metabolite with complex effects; under normal conditions, butyrate supports epithelial integrity and modulates immune responses to prevent pathogen overgrowth [26]. However, disruption of mucosal barriers, alterations in gut microbiota composition, or compromised host immunity can facilitate translocation from the gastrointestinal tract and lead to opportunistic infections. Reported clinical cases most frequently involve bacteremia, intra-abdominal infections, and abscess formation, particularly in patients with underlying gastrointestinal disease, malignancy, recent abdominal surgery, or immunosuppression [4,14,18]. Differentiating contamination or transient bacteremia from true infection may be challenging, as *Butyricimonas* spp. are fastidious organisms, difficult to culture, and often identified only through molecular techniques [17]. When invasive disease occurs, haematogenous spread can result in systemic manifestations, including sepsis and deep tissue infections, although such cases remain rare [4,9]. The increasing recognition of *Butyricimonas* spp. in clinical specimens underscores their emerging role as opportunistic pathogens and the importance of considering gut-derived anaerobes in patients presenting with unexplained bacteremia or intra-abdominal infections, particularly in the setting of mucosal disruption or immune dysfunction.

In regard to potential risk factors, history of malignancy as well as active malignancy emerged as the most common predisposing underlying conditions. Gastrointestinal (GI) cancer was the main primary lesion in the majority of these cases. GI tumors and their associated treatments disrupt the normal balance of microbes and cause gut *dysbiosis*. This is well documented in colorectal and other digestive cancers, where tumor growth correlates with shifts in microbial composition and diversity. Tumor-associated changes include loss of beneficial bacteria species and an increase in opportunistic species, enabling less abundant bacteria such as *Butyricimonas* or other anaerobes to grow beyond their usual niches [27]. Moreover, GI neoplasms may influence gut microbiota by altering the intestinal barrier integrity, allowing microbes to translocate across the mucosa, and by modifying local immune surveillance, as tumor microenvironments often suppress effective immune responses [28]. In tumor environments, butyrate can have immunomodulatory effects that may paradoxically suppress anti-tumor immunity by expanding regulatory T-cells, potentially allowing bacteria associated with these effects to persist. This has been hypothesized in microbial studies of postoperative recurrence in esophageal cancer, where *Butyricimonas* was more abundant in patients with poorer outcomes [29]. Although this does not directly equate to infection, it highlights how metabolite-mediated immune changes in cancer can alter host–microbe dynamics in favor of bacteria that might invade when barriers are compromised. In general, patients with GI tumors often have impaired immunity due to the underlying malignancy, systemic inflammation, malnutrition, or treatment. This immunocompromised state increases susceptibility to opportunistic infections from gut bacteria [4].

Recent surgical procedures, as well as obesity, also emerged as potential risk factorsin the present review. A key mechanism by which surgery predisposes to infections by gut bacteria, including rare anaerobes like *Butyricimonas*, is bacterial translocation due tointestinal barrier disruption. Major abdominal or gastrointestinal operations often involve manipulation of the bowel, disruption of normal mucosal integrity, and local inflammation. These changes can weaken the physical and immunological defenses that normally confine gut bacteria within the lumen; as a result, endogenous gut microbes can translocate across the intestinal wall into sterile compartments such as the bloodstream or peritoneal space [30]. In addition, perioperative stress, anesthesia, altered blood flow, fasting, bowel preparation, and postoperative ileus may rapidly change the composition and diversity of the gut microbiota [31]. In the present review, one case was subjected to pelvic lymph node removal for prostate cancer staging 16 days prior to infection, a different patient was subjected to aortic aneurysm surgery 24 days prior to infection while a third individual underwent Whipple procedure for adenocarcinoma of the duodenum 18 days before infection development [4,12,17]. Concerning obesity, gut dysbiosis is also the main underlying mechanism leading to *Butyricimonas* infections. Obese patients frequently exhibit altered gut microbiota composition compared with lean individuals. This includes changes in the relative abundances of bacterial taxa, reduced overall microbial diversity, and enrichment of certain bacteria capable of promoting inflammation or disrupting normal gut flora [32]. A well-documented feature of obesity is increased intestinal epithelial permeability; microbiota in obesity can directly weaken tight junction proteins that maintain epithelial integrity, increasing the likelihood of bacterial translocation [33]. Finally, obesity is associated with systemic, chronic low-grade inflammation. Adipose tissue secretes pro-inflammatory cytokines that contribute to a persistent inflammatory state that might compromise local and systemic immune responses, reducing effective clearance of microbes that penetrate the gut barrier [34].

Accurate identification of *Butyricimonas* species is challenging since advanced molecular tools, including genetic sequencing, are not routinely available in most clinical microbiology laboratories. The initial step in diagnosing *Butyricimonas* spp. infections typically involve routine blood or tissue cultures; however, microbiological identification of *Butyricimonas* is often challenging. The organism is a strictly anaerobic, Gram-negative bacillus that grows slowly and may require prolonged incubation under anaerobic conditions [4]. In culture, colonies are generally small, non-pigmented to pale gray, and lack distinctive morphological features. Because of these nonspecific growth characteristics, *Butyricimonas* spp. may be misidentified as other anaerobic Gram-negative bacteria, such as *Bacteroides* or *Prevotella* species, particularly when molecular identification methods are unavailable [16]. Precise identification of this bacterial species depends increasingly on molecular approaches, such as MALDI-TOF MS and genome sequencing. In this review, 16s rRNA and MALD TOF-MS were the methods most commonly employed to identify *Butyricimonas* species. 16S rRNA gene sequencing provides definitive species-level identification by comparing highly conserved ribosomal sequences to reference databases [10]. Similarly, MALDI-TOF MS has emerged as a rapid and effective tool for detecting *Butyricimonas* spp., although correct identification depends on the presence of validated reference spectra in the instrument’s library [17]. In clinical practice, these methods are increasingly used in combination to overcome limitations of traditional culture-based diagnostics and ensure accurate recognition of this rare opportunistic pathogen [11]. Of note, a combination of these techniques successfully diagnosed 23% of *Butyricimonas* cases. Given the expense and limited availability of these advanced molecular diagnostics, accurate identification of the pathogen should combine both biochemical testing and observation of characteristic culture features. In all reported cases of this review, cultures of clinical specimens were performed, providing data on colony morphology and biochemical behavior. Consequently, a positive culture may serve as an initial laboratory indicator, prompting subsequent confirmation using advanced molecular methods.

Standardized susceptibility criteria for *Butyricimonas* spp. have yet to be defined, due to the limited data on their antimicrobial sensitivity. Consequently, the antimicrobial susceptibility data presented in this review largely derive from individual case reports. Most *Butyricimonas* isolates reported in clinical case series have shown susceptibility to commonly used empiric anaerobic antibiotics, such as metronidazole, clindamycin, piperacillin-tazobactam, ceftriaxone, and carbapenems, which are typical agents used for anaerobic Gram-negative bacilli [7]. Strain variability has also been observed as some *Butyricimonas* isolates may be susceptible to β-lactams, including penicillin derivatives and amoxicillin–clavulanic acid, while others demonstrate resistance [7,16]. Direct data on specific resistance genes in *Butyricimonas* species are not well characterized in published case reports, largely due to the rarity of isolations and the lack of comprehensive genomic analysis in the majority of cases. However, resistance observed is consistent with mechanisms seen in other anaerobic gut bacteria, such as β-lactamase production and alterations in penicillin-binding proteins [10]. In a reported case by Whitehill et al., antimicrobial susceptibility testing of a *Butyricimonas paravirosa* clinical isolate demonstrated sensitivity to metronidazole and meropenem, while resistance was observed to ampicillin and clindamycin. Genomic analysis identified the presence of the resistance determinants *erm(F)* and *tet(Q)*, genes commonly encountered among Gram-negative anaerobic bacteria, including *Bacteroides* species. The *erm(F)* gene encodes a 23S rRNA methyltransferase that mediates resistance to macrolides, lincosamides, and streptogramin B antibiotics through post-transcriptional modification of the ribosomal target, accounting for the observed lack of clindamycin activity. In addition, *tet(Q)* confers protection against tetracycline by altering ribosomal structure, thereby preventing effective antibiotic binding [7]. The isolates in this review were, in general, susceptible to carbapenems and aminopenicillins with β-lactamase inhibitors, but exhibited resistance to several antimicrobial agents, including penicillin, aminopenicillins, colistin, and clindamycin. Table 4 summarizes all resistance patterns documented in each of the included studies. Antimicrobial resistance mechanisms in these pathogens remain largely undefined, emphasizing the need for further study as well as for the establishment of standardized criteria to guide susceptibility testing and interpretation.

Management of *Butyricimonas* infections is not defined by formal clinical guidelines, and therapeutic decisions are instead guided by case reports and anaerobic infection principles [15]. Guidance of antimicrobial therapy should rely on susceptibility testing, with emphasis on agents that combine low MICs with favorable pharmacokinetics to ensure adequate penetration into infected tissues. There have been reported cases where treatment with ceftriaxone combined with metronidazole has been successful, underscoring the value of covering anaerobic Gram-negative bacteria with a broad-spectrum β-lactam plus a nitroimidazole when feasible [7]. In instances of invasive disease complicated by abscess or septic progression, surgical drainage and source control alongside targeted antimicrobial therapy have been critical to clinical improvement. In case susceptibility testing reveals resistance to first-line agents such as penicillin or β-lactam/β-lactamase inhibitor combinations, it may be beneficial to switch to a carbapenem; these agents have been documented to lead to rapid clinical response [15]. In a case with peritoneal dialysis–related peritonitis due to *B. virosa*, prolonged courses of piperacillin-tazobactam and carbapenemshave been administered with clinical success, although data remain limited [9]. Given the absence of standardized breakpoints for *Butyricimonas*, antimicrobial selection relies on susceptibility profiles of the isolate and broader anaerobic susceptibility patterns, with empirical coverage tailored to include agents with reliable activity against anaerobic Gram-negative organisms [10]. The duration of antibiotic treatment is determined by the severity of the clinical disease. In this review, reported treatment courses varied widely, ranging from as short as 7 days to 3 months, particularly in cases of severe invasive infection [7,14].

Infections induced by *Butyricimonas* spp. are frequently associated with severe systemic illness; however, the present analysis demonstrates a relatively low mortality rate among the affected cases. In particular, only one death was attributed to the infection and concerned an individual with substantial underlying comorbidities and severe complications such as multi-organ failure and shock. The poor outcomes observed are commonly linked to delays in establishing a correct diagnosis, often due to misidentification of the organism as another Gram-negative bacterial species [18].

Several limitations should be acknowledged. The search strategy employed may not have captured the full body of published literature on epidemiology and mortality, as relevant studies could have been overlooked. In addition, the findings are derived exclusively from case reports and small case series, rendering the analysis dependent on the completeness and reliability of the original reports. Moreover, incomplete or inconsistent reporting in some publications constrained the depth of data synthesis. The small number of reported cases, which reflects the extreme rarity of documented *Butyricimonas* spp. infections in humans may preclude robust statistical analysis; nevertheless, the inclusion of all available cases provides the most comprehensive overview currently possible. Lastly, the inclusion of only English-language articles may have resulted in selection bias; however, few studies appear to have been excluded on this basis.

## 4. Materials and Methods

### 4.1. Search Strategy and Inclusion and Exclusion Criteria

This article provides a descriptive synthesis of all reported cases of infections caused by *Butyricimonas* species in humans. The main goal was to characterize the epidemiology and mortality of these infections as well as to further assess antimicrobial susceptibility patterns. Additional objectives were to collate information on underlying risk factors, clinical presentations, microbiological characteristics, and therapeutic approaches. Two investigators (A.Z. and A.G.) independently performed a comprehensive literature search using the PubMed/Medline and Scopus databases, including all publications available up to 12 January 2026. Data were collected using a predefined and standardized extraction framework. The search strategy incorporated keywords such as “Butyricimonas” in combination with the terms “infection,” “bloodstream,” “pneumonia,” “osteomyelitis,” “peritonitis,” “meningitis,” or “endocarditis.” Study selection was performed in a stepwise manner, and disagreements during the study selection process were settled by consensus with a senior author (P.I.). Studies were eligible if they involved human subjects and consisted of original investigations, such as case reports, case series, or cohort studies reporting epidemiological characteristics or clinical outcomes of *Butyricimonas*-related infections. Only English-language publications were included. Reviews, systematic reviews, experimental animal studies, and reports without full-text availability were excluded. To ensure completeness, the bibliographies of all selected articles were manually reviewed to identify additional pertinent studies.

### 4.2. Data Extraction and Definition

From each eligible publication, key variables were abstracted using a standardized data collection form, including the year of release, type of study, geographic location, and baseline patient characteristics such as age and biological sex. Information was also gathered on pre-existing health conditions, the nature and progression of the infection, covering clinical manifestations, duration of illness, and any reported complications, as well as laboratory findings related to species identification, antimicrobial susceptibility profiles, treatment regimens, and final patient outcomes. Outcome classification, including infection-attributable mortality, was recorded as defined by the original study authors.

## 5. Conclusions

This narrative review represents the first integrated overview of the epidemiological trends, clinical presentations, microbiological features, antimicrobial susceptibility profiles, therapeutic approaches, and patient outcomes reported in infections caused by *Butyricimonas* species, thereby addressing a significant gap in the existing literature. Particular emphasis is placed on underscoring the disease-causing potential of this comparatively neglected bacterialgenus. Across the reviewed cases, *B. virosa* emerged as the most common species identified, with bloodstream involvement being the most commonly reported site of infection. The isolates demonstrated variable resistance to multiple antimicrobial agents, complicating treatment decisions. Although standardized management recommendations are currently unavailable, carbapenems and piperacillin/tazobactam were the agents most frequently administered. Early initiation of antibiotics, ideally informed by in vitro susceptibility testing, appears to be a key determinant of favorable outcomes. Given the opportunistic behavior of *Butyricimonas* spp. and the diagnostic difficulties arising from limitations of routine identification platforms, heightened vigilance among clinicians and laboratory personnel is essential for accurate diagnosis and effective treatment. In spite of its limitations, this review underscores the urgent need for well-designed prospective and controlled studies to better characterize *Butyricimonas* infections and to inform the development of evidence-based therapeutic guidelines.

## Figures and Tables

**Table 1:** Characteristics of patients with *Butyricimonas* species infections.

Characteristic	All patients (*n* = 14)	Survived (*n* = 10) *	Died (*n* =2) *
Age, years, mean (SD)	43.62 (47.25–85.67)	65.25 (46.18–84.3)	72
Male gender, *n* (%)	10 (71.4)	8 (80)	2
Predisposing factors **			
Malignancy History, *n* (%)	4/13 (33.8)	3 (30)	1
Active Malignancy, *n* (%)	3/13 (23)	2 (20)	1
Previous surgery ***, *n* (%)	3/13 (2)	2 (20)	1
Obesity, *n* (%)	2/13 (15.4)	2 (20)	0
Immunosuppression, *n* (%)	2/13 (15.4)	1 (10)	1
Polymicrobial infection, *n* (%)	5 (35.7)	4 (40)	1
Clinical characteristics			
Fever, *n* (%)	5/10 (50)	3/7 (42.9)	2
Organ dysfunction, *n* (%)	5/12 (41.6)	3 (30)	2
Shock, *n* (%)	5/12 (41.6)	3 (30)	2
Treatment			
Carbapenem, *n* (%)	6/12 (50)	4 (40)	
Piperacillin/Tazobactam, *n* (%)	5/12 (41.6)	5 (50)	0
Metronidazole, *n* (%)	4/12 (33.3)	4 (40)	0
Cephalosporin, *n* (%)	3/12 (25)	3 (30)	0
Outcomes			
Deaths due to infection, *n* (%)	1/12 (8.3)	NA	NA
Deaths overall, *n* (%)	2/12(16.6)	NA	NA

*: no data on clinical outcome wereavailable for twopatients, **: data on risk factors werenot available for onepatient, ***: within the past 3 months, NA: not applicable.

**Table 2:** Association between infection site and common clinical complications.

Study/Year	Infection Sites	Clinical Complications
Whitehill et al., 2024 [7]	Bacteremia, GI tract	Fever, Organ dysfunction
Lau et al., 2022 [9]	Peritonitis	Septic Shock
Ogawa et al., 2018 [11]	Bacteremia, Peritonitis	Septic Shock, Organ dysfunction
García-Agudo et al., 2018 [13]	Bacteremia, GI tract	Fever
Wessendorf et al., 2024 [15]	Skin/Soft tissue	Septic Shock, Organ dysfunction
Ulger Toprak et al., 2015 [4]	Bacteremia	Fever, Septic Shock, Organ dysfunction
Mehta et al., 2015 [17]	Bacteremia	Fever
Gasos et al., 2019 [18]	Bacteremia, Skin/Soft tissue	Fever, Hypovolemic Shock, Organ dysfunction

GI: Gastrointestinal.

**Table 3:** Antimicrobial Resistance Profiles.

Antimicrobial Agent	Number of Patients	Resistance (%)
Clindamycin	4/7	57.1
Penicillin	4/5	80
Aminopenicillin	3/5	60
Colistin	3/3	100
Cephalosporins	2/2	100
Vancomycin	2/3	66.7
Aminoglycosides	2/3	66.7
Piperacillin-Tazobactam	1/5	20
Metronidazole	1/8	12.5
Tetracyclines	1/2	50
Oxacillin, Macrolides, Quinolones, Fosfomycin	1/1	100

**Table 4:** Antimicrobial resistance was documented among the included studies.

Study/Reference	Species	Antimicrobial Resistance
Whitehill et al. [7]	*B. paravirosa*	Aminopenicillin, Tetracycline, Clindamycin
Kamel et al. [8]	*B. faecihominis*	Aminopenicillin, Clindamycin
Lau et al. [9]	*B. virosa*	Aminopenicillin, Clindamycin, Metronidazole
Enemchukwu et al. [10]	*B. virosa*	Penicillin, Cephalosporin
Ogawa et al. [11]	*B. virosa*	NR
De Donder et al. [12]	*B. virosa*	NR
García-Agudo et al. [13]	*B. virosa*	Penicillin
Ferry et al. [14]	NR	NR
Wessendorf et al. [15]	*B. faecihominis*	Penicillin, Piperacillin/Tazobactam
Togo et al. [16]	*B. phoceensis*	Oxacillin, Colistin, Cephalosporin, Quinolone, Clindamycin, Macrolide, Fosfomycin
Ulger Toprak et al. [4]	*B. virosa*	Colistin, Aminoglycoside, Vancomycin
Bordigoni et al. [3]	*B. vaginalis*	NR
Mehta et al. [17]	*B. virosa*	Colistin, Aminoglycoside, Vancomycin
Gasos et al. [18]	*B. virosa*	Penicillin

NR: Not reported.

## Data Availability

No new data were created or analyzed in this study. Data sharing is not applicable to this article.

## Supplementary Materials

The following supporting information can be downloaded at: https://www.mdpi.com/article/10.3390/antibiotics15030297/s1, Table S1: Characteristics of all included studies.

## Abbreviations

The following abbreviations are used in this manuscript:

MALDI-TOF MS	Matrix-Assisted Laser Desorption/Ionization Time-Of-Flight Mass Spectrometry
GI	Gastrointestinal
NR	Not reported
TMP-SMX	Trimethoprim-Sulfamethoxazole
