# Supplementary material for: From Gut Commensal to Opportunistic Pathogen: A Narrative Review of Butyricimonas Infections in Humans
Source: Antibiotics (Basel). 2026 Mar 14;15(3):297. doi: 10.3390/antibiotics15030297 (PMC13024695; doi:10.3390/antibiotics15030297)
Supplement: Supplementary file 1 [file antibiotics-15-00297-s001.zip › antibiotics-4144915-supplementary.pdf]

Table S1. Characteristics of all included studies *(NR: Not Reported, M: Male, F: Female, ESRD: end-stage renal disease, PD: peritoneal dialysis, ISx: Immunosuppression, T2DM: Type 2 Diabetes Mellitus, GI: gastrointestinal, TMP-SMX: Trimethoprim-Sulfamethoxazole, *: surgery within the past 3 months*

Author name/ Reference number	Year	Age (years)	Gender	Comorbidities	Type of Infection	Antimicrobial Resistance	Antimicrobial Treatment	Fatality
Whitehill et al. (7)	2024	94	F	History of breast malignancy	Bacteremia, terminal ileitis	Aminopenicillin, Tetracycline, Clindamycin	Piperacillin/Tazobactam, Cephalosporin, Metronidazole	-
Kamel et al. (8)	2021	38	F	-	Bacteremia, appendicitis, peritonitis	Aminopenicillin, Clindamycin	Aminopenicillin/ clavulanic	-
Lau et al. (9)	2022	65	M	T2DM, ESRD on PD	Peritonitis	Aminopenicillin, Clindamycin, Metronidazole	Piperacillin/Tazobactam, Cephalosporin, Carbapenem, Aminoglycoside	-
Enemchukwu et al. (10)	2016	69	M	-	Bacteremia, diverticulitis	Penicillin, Cephalosporin	Cephalosporin, Quinolone, Metronidazole	-
Ogawa et al. (11)	2018	68	M	History of colon malignancy, Active prostate malignancy, recent surgery*	Bacteremia, peritonitis	NR	Carbapenem	-
De Donder et al. (12)	2020	78	M	-	Necrotizing fasciitis	NR	Piperacillin/Tazobactam, Vancomycin	-
García-Agudo et al. (13)	2018	90	M	Obesity, Trauma	Bacteremia, appendicitis	Penicillin	Piperacillin/Tazobactam, TMP-SMX, Metronidazole	-
Ferry et al. (14)	2015	30	M	Trauma	Bone/ joint infection	NR	Carbapenem, Clindamycin, Metronidazole	-
Wessendorf et al. (15)	2024	50	M	-	Skin/ Soft tissues	Penicillin, Piperacillin/	Piperacillin/Tazobactam, Carbapenem	-

						Tazobactam		
Togo et al. (16)	2016	57	F	Obesity	GI infection	Oxacillin, Colistin, Cephalosporin, Quinolone, Clindamycin, Macrolide, Fosfomycin	NR	NR
Ulger Toprak et al. (4)	2015	72	M	History of colon malignancy, recent surgery*	Bacteremia	Colistin, Aminoglycoside, Vancomycin	Carbapenem, Colistin	+
Bordigoni et al. (3)	2020	NR	F	NR	Vaginosis	NR	NR	NR
Mehta et al. (17)	2015	81	M	Active duodenum malignancy, recent surgery*	Bacteremia	Colistin, Aminoglycoside, Vancomycin	No antibiotics	-
Gasos et al. (18)	2019	72	M	History of colon malignancy	Bactreremia, Skin/ Soft tissues	Penicillin	Carbapenem	+
